# Learning to decipher time-compressed speech: Robust acquisition with a slight difficulty in generalization among young adults with developmental dyslexia

**DOI:** 10.1371/journal.pone.0205110

**Published:** 2018-10-24

**Authors:** Yafit Gabay, Avi Karni, Karen Banai

**Affiliations:** 1 Department of Special Education, University of Haifa, Haifa, Israel; 2 Edmond J. Safra Brain Research Center for the Study of Learning Disabilities, Department of Learning Disabilities, University of Haifa, Haifa, Israel; 3 Sagol Department of Neurobiology, University of Haifa, Haifa, Israel; 4 Department of Communications Sciences and Disorders, University of Haifa, Haifa, Israel; French National Center for Scientific Research (CNRS) & University of Lyon, FRANCE

## Abstract

Learning to decipher acoustically distorted speech serves as a test case for the study of language-related skill acquisition in persons with developmental dyslexia (DD). Deciphering this type of input is rarely learned explicitly and does not yield conscious insights. Problems in implicit and procedural skill learning have been proposed as possible causes of DD. Here we examined the learning of time-compressed (accelerated) speech and its generalization to novel materials among young adults with DD compared to typical readers (TD). All participants completed a training session that involved judging the semantic plausibility of sentences, during which the level of time-compression was changed using an adaptive (staircase) procedure according to each participant’s performance. In the test, phase learning (test on same items) and generalization (test on new items and same items spoken by a new speaker) were assessed. Both groups showed robust gains after training. Moreover, after training, the initial disadvantage of the DD group was no longer significant. After training, both groups experienced relative difficulties in deciphering learned tokens spoken by a different voice, though participants with DD were less able to generalize the gains to deciphering new tokens. Thus, DD individuals benefited from repeated experience with time-compressed speech no less than typical readers, but their evolving skill was apparently more dependent on the specific characteristics of the tokens. Atypical generalization, which indicates that perceptual learning is contingent on lower-level features of the input though does not necessarily point to impaired learning potential per se, may explain some of the contradictory findings in published studies of speech perception in DD.

## Introduction

Although usually transparent to listeners, speech perception is quite a challenging task. In particular, it requires mapping the acoustic input onto stable (pre-lexical/lexical) representations even though the speech signal itself is variable as a result of between-speaker differences, changes in speech rate [1] and environmental conditions [2]. Speech stimuli constitute a learning challenge for the perceptual system because accurate speech recognition requires generalization across the highly variable acoustic information that underlies the speech signal. Listeners are capable of overcoming these variations in speech through perceptual learning, according to which they align their perceptual system with new variations in the speech input [3]. Perceptual learning has been demonstrated across a variety of tasks in which the speech signal is noisy, distorted (e.g., noise vocoded, spectrally shifted or time-compressed speech) or otherwise unusual (e.g., unfamiliar dialects or accents) [4]. Previous studies suggest that adaptive training procedures that start off with relatively little signal distortion (“easy” items, not far removed from standard speech) may be advantageous for learning and its generalization [5, 6].

### Implicit and procedural learning in speech perception

Since speech is rarely learned explicitly and perceptual learning does not yield conscious insights that can be easily communicated, the perceptual learning of speech is a case of implicit learning of skills that are essential to human communication [7]. Implicit learning refers to situations in which learning occurs incidentally [8], and the knowledge gained through this process is believed to be implicit as participants find it difficult to conceptualize what has been learned [9]. Implicit and procedural learning has been related to the acquisition and formation of motor skills [10]. An accumulating body of evidence also implicates its involvement in language-related skills, including the acquisition of grammar, syntax, morphology and phonology [11–14]. Research closely related to the present study also implicates implicit learning in the perceptual learning of speech [15].

### Implicit and procedural learning in developmental dyslexia

Developmental Dyslexia (DD) is one of the most common neurodevelopmental disorders, with prevalence rates estimated at 5%-10% [16]. Despite extensive research, the underlying biological and cognitive causes of DD remain unclear. DD has been thought to arise from phonological impairments [17]. Recent conceptualizations of dyslexia implicate domain-general procedural and/or implicit learning systems in its etiology [13, 18–22]. These views are based on increasing evidence for the role of non-declarative systems in language learning and development [11–15] and on the plethora of findings that individuals with dyslexia often demonstrate impairments on procedural and implicit learning tasks [21, 23–31]. Although there is evidence suggesting intact procedural learning in DD [32–34], a recent meta-analysis argues in favor of the possibility that compensatory declarative learning mechanisms may mask procedural learning deficits in DD [27].

Although perceptual learning has been examined previously in individuals with DD in both the visual [35, 36] and the auditory modalities [37–42], perceptual learning of speech stimuli has rarely been assessed. Compared with other stimuli, speech stimuli represent a different learning challenge for the perceptual system. Generalization, for example, may be particularly important for speech perception due to the highly variable nature of the acoustic information that underlies the speech signal. The goal of the current study was therefore to investigate the perceptual learning of distorted speech in people with DD. Such speech is often particularly challenging for people with dyslexia [43].

Many studies suggest that typically developing individuals can adapt to such speech quite rapidly, especially under favorable learning conditions [44]. Recent studies suggest that adaptive protocols that begin with easy tasks provide such conditions [5, 6]. Previous observations suggest that adaptive training conditions yield more perceptual learning and generalization than constant training conditions [5]. Thus, to provide a strict test of the hypothesis that learning may differ between DD and TD participants, we used an adaptive protocol in the current study. Three indices of learning were investigated. First we asked whether rapid baseline adaptation to time-compressed speech is affected by DD. Second, we compared the effects of adaptive training on the recognition of time-compressed speech between the two groups of readers. Third, we compared the ability to transfer the training-related gains to novel conditions, i.e., conditions not encountered in training, across the two groups of readers. Two types of transfer were studied: (1) transfer to stimuli that share the high-level features of the trained tokens, but differ in their low-level features, i.e., sentences identical to those presented during training but produced by a new unfamiliar speaker; (2) transfer to stimuli that share the low-level features of the trained stimuli but differ in their high-level features, i.e., novel sentences but uttered by the speaker encountered in the training phase.

## Methods

### Participants

Participants were 24 university students (undergraduates or graduate students), among them 12 dyslexics (5 female) and 12 typical readers (7 female). A similar sample size was sufficient to detect group differences on the same task between native and non-native listeners [45]. Participants were native Hebrew speakers with no history of neurological disorders, psychiatric disorders or attention deficits. In addition, participants were right handed, had normal or corrected-to-normal vision, and normal hearing (participants in the DD group were screened for normal hearing; participants in the control group declared they had no hearing impairment). The DD group was recruited from the Student Support Service at the University of Haifa, a center that provides support for students with learning disabilities. Dyslexia was diagnosed by the University of Haifa Learning Disabilities Diagnostic Center by means of the MATAL test. This test is designed to assess developmental disabilities (Dyslexia, Dysgraphia, Dyscalculia, and Attention Deficit Disorder) in adults who are native Hebrew speakers. The MATAL is a standardized test developed by the Israeli National Institute for Testing and the Israeli Council for Higher Education [46]. The test consists of 20 tests and 54 performance measures, and was validated and normed with a standardization sample of 508 participants. The MATAL has been used in many previous investigations for the assessment of dyslexia [47, 48]. The typical reading group (TD) consisted of participants with no history of learning disabilities. Both the DD and the TD groups performed a battery of cognitive and literacy tests similar to the battery used in the study by]. The ethics committee of the Faculty of Social Welfare and Health Sciences at the University of Haifa (199/12) approved all aspects of the study and written informed consent was obtained from all participants.

### Cognitive and literacy measures

#### Intellectual ability

Intelligence was assessed by means of two subtests from the Wechsler Intelligence test for adults [49]. One is the non-verbal block design task in which participants are required to rearrange blocks with different color patterns according to a stimulus presented to them upon a card. The other is the verbal similarities subtest in which participants are required to indicate what two words in a pair have in common (i.e., what do dog and cat have in common = both are animals).

#### Verbal working memory

Verbal working memory was assessed by the Digit Span subtest from the Wechsler Adult Intelligence Scale [49]. In this test the examiner reads a list of digits to the examinee and the examinee is required to repeat the digits in that order (forward) or to state the digits in reverse order (backward). Task administration is stopped after failure to recall on two trials with a similar number of digits.

#### Reading skills

Decoding, reading fluency, and reading comprehension tests were administered, as described in the following sections.

Two tests were used to assess decoding skills: One Minute Tests of Words [50] and of Non-words [51], which examine the number of words and non-words accurately read aloud within a time limitation of one minute. The first test included 168 non-vowelized words of an equal level of difficulty listed in columns. The second test was composed of 86 successively difficult vowelized non-words listed in columns. In both tests, measures of accuracy (number of correct words read per minute) and of speed (number of items read per minute) were collected.

The Oral Reading Tests obtained from the reading comprehension subset of the Israeli Psychometric Exam was used to assess reading fluency. In this test, participants were required to read a text of 216 words aloud, as quickly and accurately as possible. The number of words read correctly per minute was calculated.

#### Reading-related skills

**Phonological awareness** was assessed by the following tests: Phoneme Deletion, Segmentation and Parsing [52]. The phoneme deletion test consists of 25 non-words. In this test, the experimenter reads a word and a phoneme aloud and the participant is required to indicate how the word sounds after deletion of this phoneme. The segmentation test includes 16 non-words that are read to the examinee by the experimenter. The task is to segment the word into its basic phonological sounds as quickly as possible. The parsing test [53] contains 46 rows of words. Each row is composed of four words printed with no spaces between them. The participants’ task was to identify the words in each row by drawing a line to mark where the spaces should be. For all tests, both accuracy (number of correct letters/objects read per minute) and time (the time participants required to complete the task) were measured.

**Naming skills** were assessed through the RAN- Naming Speed Test [54] that consists of the following tests for naming objects and letters and for naming alternating objects and letters. In the letter naming test (RAN letters), five (non-final) Hebrew letters—ס, א, ד, ג, ל—were repeatedly presented in random order, with each letter repeated ten times. The participants were asked to read the 50 letters aloud as quickly and accurately as they could. The object naming test (RAN object) consists of pictures of five objects: flower, cat, book, watch and flag, where each object is repeated randomly 10 times. The participants were asked to name the 50 pictures aloud as accurately and quickly as they could. In both tasks, the accuracy rates and the time for naming the entire list were measured.

TD and DD listeners did not differ in intelligence (as measured by the block design subtest and by verbal ability scores measured by the similarities subtest) or chronological age. However, there were significant group differences with regard to reading, naming and phonological skills (see Table 1), confirming group assignments with respect to reading ability.

**Table 1:** Performance of the DD and TD groups on cognitive and literacy measures.

	Group					
Measure	*Dyslexia* *Mean (SD)*	*Range*	*Control* *Mean (SD)*	*Range*	*t value*	p
Age (in years)	27 (2.21)	24–35	27.75 (2.98)	25–32	-.*69*	.49
**Decoding**						
Oral word recognition accuracy	63.58 (17.84)	39–98	119.58 (15.24)	98–153	-8.04	.01
Oral words recognition speed	67.77 (16.53)	45–98	119.08 (15.24)	98–153	-8.15	.01
Oral non-words recognition accuracy	26.08 (9.69)	9–38	60.41 (11.42)	45–81	-7.93	.01
Oral non-words recognition speed	39.83 (9.03)	29–64	68 (11.81)	50–86	-6.55	.01
**Reading Fluency measures**						
Oral text fluency- words per min	96.05 (33.15)	14–145	164.01 (20.42)	127–212	-5.84	.01
Naming digits	28.38 (5.13)	20–37	20 (1.95)	16–23	-5.28	.01
Naming objects	43.81 (4.82)	38–51	31.08 (3.39)	27–39	7.46	.01
**Phonological processing**						
Phoneme deletion (time)	194.84 (60.64)	111–293	105.16 (22.5)	81–157	4.8	.01
Phoneme deletion (accuracy)	19.33 (6.3)	3–25	23.66 (2.96)	15–25	19.33	.05
Segmentation (time)	120.8 (59.06)	88–304	94.83 (42.29)	55–205	1.23	n.s.
Segmentation (accuracy)	11 (4.22)	2–16	14.33 (2.14)	9–16	-2.43	.05
Parsing (time)	329.36 (73.88)	192–244	178.25 (32.06)	135–232	6.48	.01
Parsing (accuracy)	42.41 (3.08)	36–46	45.08 (.79)	44–46	-2.89	.01
Digit Span	8.5 (1.83)	6–11	12.33 (2.64)	9–19	-4.13	.01
**Intellectual ability**						
Block design (nonverbal intelligence)	11.66 (3.39)	4–16	12.5 (2.43)	8–16	-0.69	n.s.
Similarities (verbal intelligence)	12.08 (1.56)	10–15	12.16 (1.69)	10–15	-0.12	n.s.

### Experimental procedure

#### Stimuli

The stimuli and the procedure were similar to those used in our previous study [5]. A young male native speaker of Hebrew (the trained speaker) recorded and sampled the stimuli at 44 kHz using a standard microphone and PC soundcard and Audacity software. Additionally, several sentences designed to assess generalization to a new speaker were recorded by a second native Hebrew speaker. RMS levels of all sentences were normalized after recording and before compression. Stimuli were time-compressed using a WSOLA algorithm [55], which changes speech rate but preserves other qualities such as pitch and timbre.

The sentences included 120 simple active subject-verb-object (SVO) sentences in Hebrew taken from the study by Prior and Bentin [56]. Each sentence contained 5–6 words and had adjectives modifying both the subject and the object. The duration of the naturally spoken sentences ranged from 2.3–4.2 s (72–144 words/minute). This speech rate is similar to that of Israeli newscasters [57]. Sixty sentences were semantically plausible (true, e.g., “The municipal museum purchased the impressionist painting”), whereas the remaining sentences (false) contained a semantic violation that rendered them improbable (e.g., “The municipal museum ate the impressionist painting”). One hundred sentences (50 true) were used for training. Twenty of those sentences were presented in the pre-test and test phases to assess learning of the repeated tokens. Likewise, 20 of the trained sentences uttered by a different speaker were used to assess cross-speaker generalization. The remaining 20 sentences were used to assess generalization to untrained tokens.

#### Procedure

Testing took place in a quiet room and participants were seated directly in front of a computer monitor during the entire experiment. Stimulus presentation and time compression manipulation were controlled by Matlab. Stimuli were presented binaurally using headphones (Sennheiser HD-215). The experiment consisted of three phases: a pre-test phase in which baseline performance was assessed, a training phase and a test phase. During the pre-test and test phases, participants were required to write down each of the presented sentences as accurately as they could. During the training phase, participants were required to press a key to indicate whether the sentences they heard were plausible or not.

The experiment was administered in one session of approximately one hour. Cognitive and literacy tests were administered to participants in a different session. During the session, participants completed the pre-test, the training and the test. The training phase consisted of 100 different time-compressed sentences. During training, listeners performed a semantic verification task on these sentences during five blocks, each containing 60 trials. After hearing each sentence, listeners were required to determine whether it was semantically improbable (false) or probable (true). Sentences were selected at random (without replacement) until all 100 sentences were presented, after which random selection began again. Visual feedback (smiling/sad face) was delivered to participants after each response. In the present study, an adaptive staircase training protocol was used. That is, training started with a compression level of 65% of the naturally spoken duration. After that, compression was adapted using a 2-down/1-up staircase procedure in 25 logarithmically equal steps to a maximal compression of 20% [58]. The considerations that led us to select the stimuli (compression rates) were very similar to considerations used in many perceptual learning studies (e.g., [59]). The idea was to start from typical levels of performance and try to push the participants’ performance as much as possible into conditions wherein untrained individuals would fail to correctly recognize the stimuli. Thus, the compression rates were chosen so as to provide experience with speech rates that range from easily recognizable up to high-speed speech stimuli that cannot be recognized by native listeners without specific training [60, 61].

#### Test and training tasks

**Fig 1:**
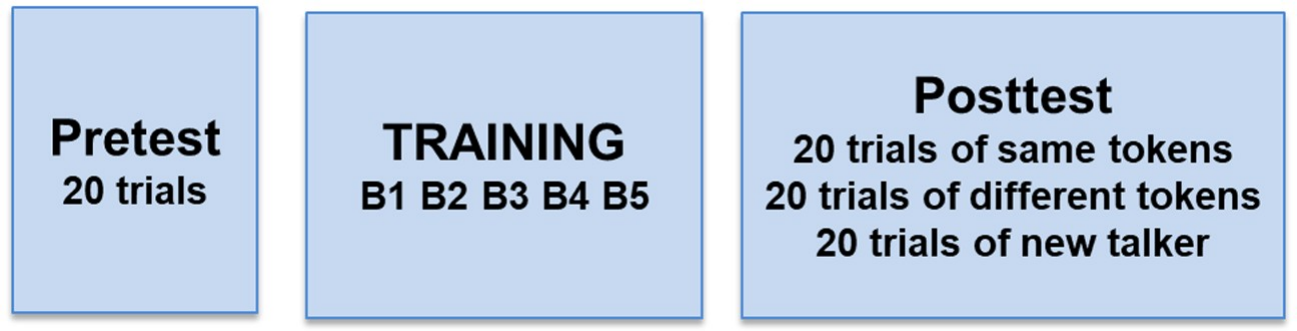
Procedure. Participants performed pre-test and five blocks of training (each contained sixty trials). After that, participants performed a test with three conditions. Test performance on same tokens is indicative of learning. Test performances with the new speaker and with new tokens are indicative of generalization.

During the pretest phase, 20 sentences compressed to 30% of their naturally spoken duration were presented. During the test phase, blocks of 20 sentences compressed to 30% of their naturally spoken duration were presented. The participants’ task was to write the sentences down as accurately as they could. The test phase consisted of three different conditions of 20 trials each (repeated items, new items, repeated items presented by a different speaker). 1) In the repeated-items test, 20 sentences randomly selected from the training set were uttered by the same male speaker from the training phase. 2) In the new-items test, 20 new sentences with similar semantic structure to those in the training phrase were uttered by the same speaker heard throughout the training phase. 3) In the test of repeated items presented by a different speaker, 20 sentences were selected from the training set but uttered by a different male speaker. The order of the three tests was counterbalanced across participants. No feedback was provided during either the pre-test or the test. See Fig 1 for an illustration of the design.

## Results

### Data analysis

Performance during the pre-test and test was quantified as the mean proportion of words correctly identified across all sentences in a given condition. Orthographic errors (e.g., homophones) were not calculated as errors because the purpose was to assess whether listeners heard the sentences correctly and not to assess their writing skills. Incomplete/incorrect suffixes were considered errors because Hebrew is an inflected language and suffixes affect the meaning of the sentence (e.g., changing the timing of an event from past to future). The mean proportion of sentences correctly judged (verification) in each block was used to quantify performance during the training phase. To this end, mean verification threshold were calculated based on the five final trials in each block.

We first calculated participants’ level of performance during the pretest phase. Previous research has contended that for typical readers rapid learning can be observed even during the pretest phase [60]. We then estimated training-phase performance in the two groups by calculating the 71% correct verification thresholds for each listener (for details see [45]. Group differences after training were then evaluated. For this purpose, test performance was compared to pre-test performance on the repeated-tokens as evidence for learning across groups. Finally, test performance on the trained items was compared to performance on new items and on items produced by a new speaker to test for generalization.

### Rapid learning during the pre-test

**Fig 2:**
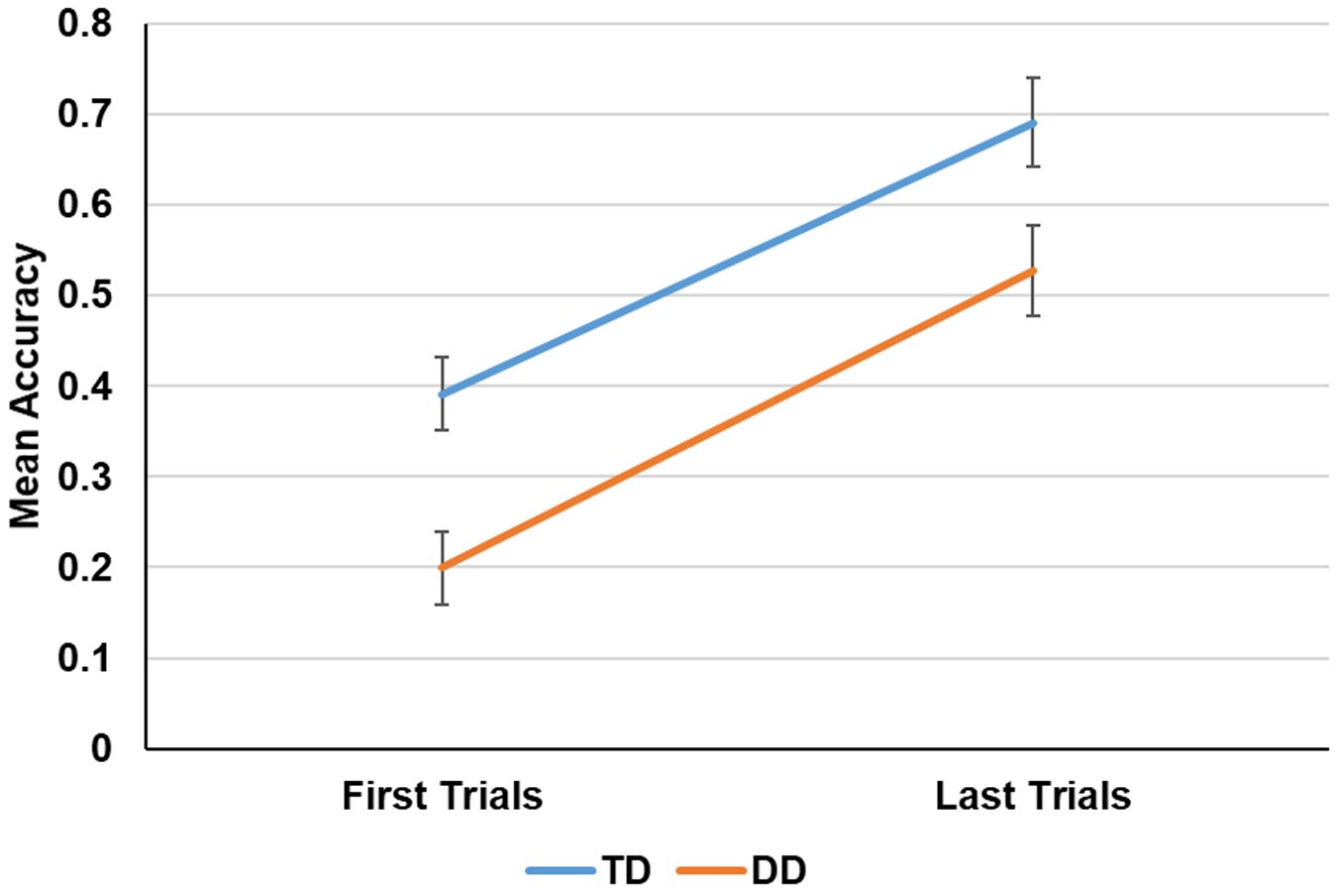
Pretest performance (mean of five first trials vs. mean of five last trials) as a function of group (TD vs. TD). Error bars show the 95% confidence interval of the mean.

Fig 2 shows the mean performance accuracy over the first and last five trials of the pretest phase. An analysis of variance was conducted, with group (TD vs. DD) as a between-subject factor, learning (first five trials vs. five last trials) as within-subject factors and mean proportion of words correctly identified as the dependent variable. The main effect of group was significant, suggesting that DD participants were less able to decipher time-compressed speech compared to TD participants (*F*(1, 22) = 8.68, *p*<.01; *η*_*p*_^*2*^ = .28). However, the main effect of learning was also significant, suggesting that recognition accuracy improved during the test (rapid learning, *F*(1, 22) = 122.58, *p*<.01; *η*_*p*_^*2*^ = .28). There was no significant interaction of group by learning (*F*<1), suggesting that both groups improved to a similar extent during this phase.

### Learning during the training phase

**Fig 3:**
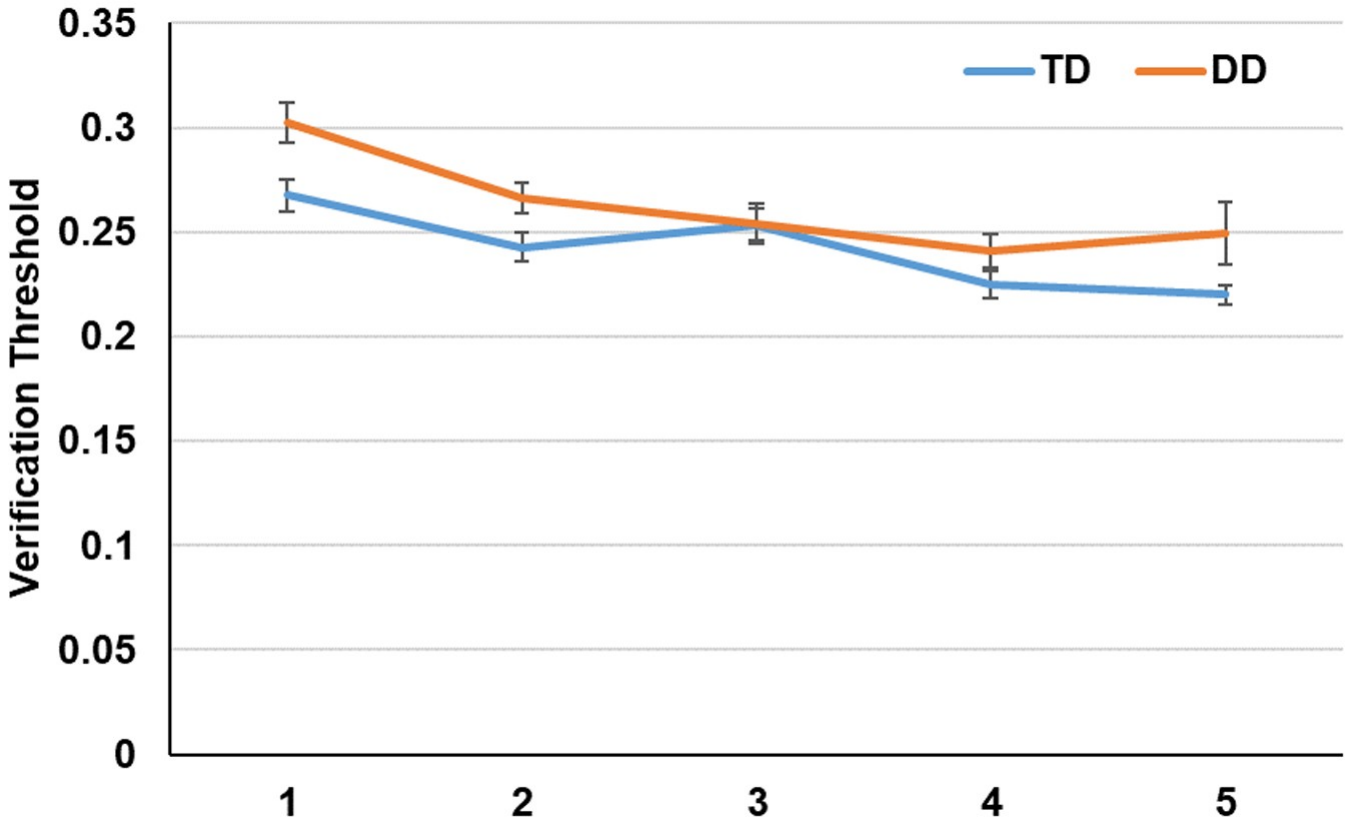
Training-phase performance as a function of group (TD vs. DD). Error bars show the 95% confidence interval of the mean.

Fig 3 depicts the performance of both the DD and the TD groups over the course of training. An ANOVA was conducted, with group (TD vs. DD) as a between-subject factor and the mean verification thresholds in each block of training (1–5) as within-subjects factors. The main effect of group was significant (*F* (1, 22) = 8.7, *p*<.01; *η*_*p*_^*2*^ = .27), indicating that TD listeners were generally able to correctly judge sentences that were more time-compressed compared to DD listeners. Nevertheless, the difference between the two groups was quite small and as can be seen in Fig 3, a single training block sufficed to bring the level of performance in the DD group up to that of the initial performance of the TD participants. The main effect of block was significant as well, suggesting that both groups improved with practice (*F* (1, 22) = 122.6, *p*<.01; *η*_*p*_^*2*^ = .84). The interaction of group by learning was not significant (*F*<1), suggesting that despite the overall performance differences, the amount of learning was similar in the two groups.

### Training-induced learning

**Fig 4:**
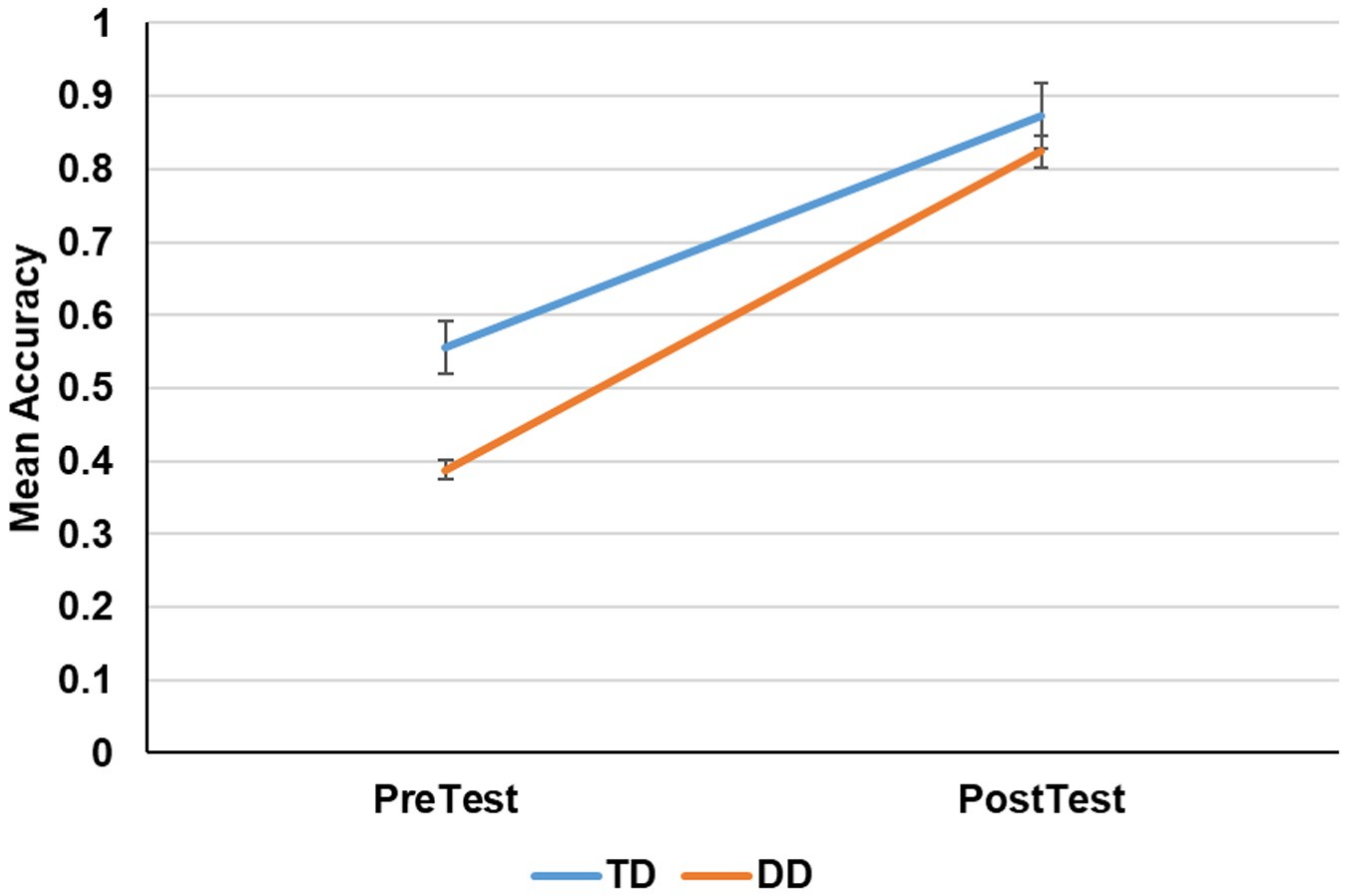
Pretest vs. test-phase performance on the repeated tokens conditions. Error bars show the 95% confidence interval of the mean.

Fig 4 shows the performance of the two groups in the repeated-tokens condition on the pre-test and test. An analysis of variance (ANOVA) on mean proportion of words correctly identified with group (DD vs. TD) as a between-subjects factor and test (pre vs. post) as a within-subjects factor yielded a main effect of group (*F* (1, 22) = 8.3, *p*<.01; *η*_*p*_^*2*^ = .27). There was a main effect of test, indicating an increase in performance accuracy by the end of training (*F* (1, 22) = 268.21, *p*<.01; *η*_*p*_^*2*^ = .92). The interaction of group by phase was also significant (*F* (1, 22) = 6.63, *p*<.05; *η*_*p*_^*2*^ = .23), reflecting the larger improvements in the DD group. TD listeners significantly outperformed DD listeners during the pre-test (*F* (1, 22) = 8.63, *p*<.01), but the test showed only a trend toward a between-group difference (*F* (1, 22) = 3.67, *p* = .065). Thus, training with time-compressed speech was helpful in reducing the performance differences between TD and DD readers.

### Training-induced generalization

**Fig 5:**
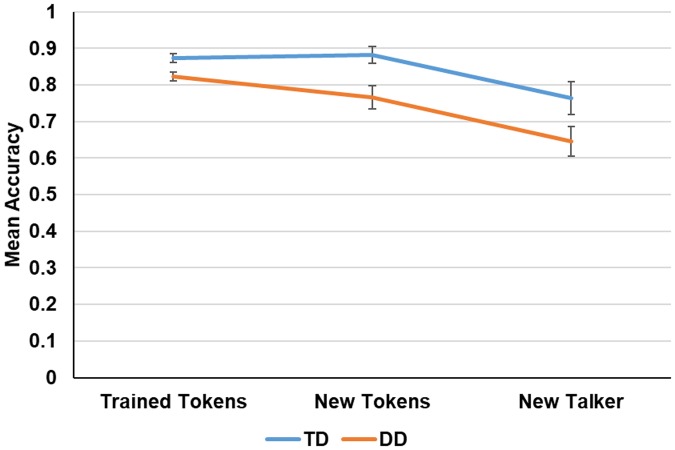
Test-phase performance on the trained tokens, new tokens and new speaker conditions as a function of group (TD vs. DD). Error bars show the 95% confidence interval of the mean.

Fig 5 shows the performance of the two groups in the trained-token condition and in the two transfer conditions. The participants’ ability to generalize the gains they acquired during training to writing (reproducing) the time-compressed sentences was compared to the participants’ ability to reproduce novel time-compressed sentences (new tokens) and separately compared to their ability to reproduce the trained tokens recorded by a different speaker (new speaker).

#### Generalization to new tokens

An analysis of variance (ANOVA) was conducted on the mean proportion of words correctly identified, with group (DD vs. TD) as a between-subjects factor and token type (trained sentences vs. new sentences) as a within-subject factor. Group exhibited a significant main effect, reflecting the lower recognition accuracy in the DD group, (*F* (1, 22) = 9.21, *p*<.01; *η*_*p*_^*2*^ = .29), while token type exhibited only a marginal effect (*F* (1, 22) = 3.79, p = .064; *η*_*p*_^*2*^ = .14), indicating that in general participants were marginally more accurate when tested with repeated tokens compared to new tokens. However, the group-by-token type interaction was significant (*F* (1, 22) = 6.88, p<.05; *η*_*p*_^*2*^ = .23; *η*_*p*_^*2*^ = .23). Further analysis revealed that whereas TD listeners recognized repeated and newly encountered items similarly (*F*<1), participants with DD were significantly less accurate with newly encountered than with repeated tokens (*F* (1, 22) = 10.44, *p*<.01). Together these findings suggest that listeners with DD were less able to transfer their learning-related gains to tokens they had not encountered before.

#### Generalization to a new speaker

An analysis of variance (ANOVA) on the mean proportion of words correctly identified was conducted, with group (DD vs. TD) as a between-subjects factor and token type (trained speaker vs. new speaker) as a within-subjects factor. There was a significant main effect of group, reflecting the less accurate performance of the DD group (*F* (1, 22) = 6.03, *p*<.05; *η*_*p*_^*2*^ = 21). The main effect of token type (speaker voice) was also significant (*F* (1, 22) = 51.71, p<.01; *η*_*p*_^*2*^ = 701), indicating that in general participants performed better during the test with the trained tokens compared to the same tokens but uttered by a new speaker. The group-by-token type interaction was not significant (*F* (1, 22) = 2.85, p = .10; *η*_*p*_^*2*^ = .11).

### Relationship between task performance and individual reading ability

In addition to the group analyses reported above, we explored the relationships between test-phase performance in the three different conditions (learning and generalization to new tokens/new speaker) and reading ability as measured by four standardized tests. As shown in Table 2, generalization to new tokens was positively correlated with the estimates of reading ability. Furthermore, a negative correlation was observed between the generalization scores and the time required to name objects and digits. Lastly, a negative correlation was observed between generalization scores (in both the new token and the new speaker conditions) and time required for parsing printed words with no space between them. No correlations were observed between the generalization scores (in both the new token and the new speaker conditions) and intellectual abilities or working memory (digit span). Given that these correlations are consistent with the findings of the primary group analyses presented above (not surprisingly, as the groups were defined based on literacy abilities) and given the relatively small sample, we refrain from any further discussion and interpretation of these findings.

**Table 2:** Relationship between task performance and individual reading ability.

Measure	*Learning*	*Generalization (new tokens)*	*Generalization (new talker)*
**Decoding**			
Oral word recognition accuracy	.179	.409*	.370
Oral words recognition speed	.110	.324	.281
Oral non-words recognition accuracy	.303	.509*	.448*
Oral non-words recognition speed	.111	.416*	.346
**Reading Fluency measures**			
Oral text fluency- words per min	-.076	-.344	-.282
Naming digits	-.418*	-.539**	-.631**
Naming objects	-.245	-.430**	-.400
**Phonological processing**			
Phoneme deletion (time)	.078	-.262	-.175
Phoneme deletion (accuracy)	-.164	.12	.037
Segmentation (time)	.080	.016	.185
Segmentation (accuracy)	.053	.145	.105
Parsing (time)	-.118	-.473**	-.432*
Parsing (accuracy)	-.127	.115	.192
Digit Span	.117	.374	.145
**Intellectual ability**			
Block design (nonverbal intelligence)	-.256	-.231	-.048
Similarities (verbal intelligence)	-.210	.078	-.024

* *p* < .05.

** *p* < .01.

## Discussion

Impairments in implicit skill acquisition have been proposed to have a deleterious impact in DD [13, 18–21, 24, 62]. Perceptual learning of speech represents a case of procedural learning (i.e., skill learning—how to, what to do knowledge—that are acquired implicitly and are difficult to verbalize explicitly, [7]. The current results show that despite the initial advantage of typical readers over struggling readers in the ability to decipher time-compressed speech, both groups improved with practice, such that the magnitude of learning was similar in the two groups. Thus, given an identical training experience in deciphering time-compressed speech, young adults with DD were as adept in acquiring the specific skill as their typical reading peers. Moreover, compared to their pre-training baseline performance, both groups improved in deciphering tokens uttered in a new (untrained) speaker’s voice and much improved in their ability to decipher new time-compressed tokens. Nevertheless, listeners with DD were less able to transfer their learning-related gains to tokens that were not encountered during the training session. Both groups were hampered in deciphering the trained tokens delivered by a new speaker compared to their ability to decipher tokens presented in the familiar (trained) voice.

Baseline recognition of time-compressed speech was less accurate among DD participants than among TD participants. This relative disadvantage is consistent with previous findings reporting that the processing of time-compressed speech is deficient among impaired readers [63, 64]. Yet although on average the performance of DD readers during the pretest phase was below that of TD readers, a single session of adaptive training with time-compressed speech resulted in a reduction of group differences. Moreover, the DD group gained as much and even more than their peers from this practice. These results are consistent with previous studies indicating that given appropriate training conditions, individuals with DD can reveal their extant, intact, perceptual learning ability in both visual [35, 36] and auditory [37, 39–42, 65] domains. Thus, no less than their peers people with DD retain the potential to benefit from practice, i.e., from repeated experience with stimuli that defy explicit awareness of what has been gained.

Yet despite relatively intact learning, the ability to generalize the gains attained in training to new tokens and a new speaker was relatively less robust in the DD group. In particular, after practice, unimpaired readers were as adept in recognizing new speech items as they were in recognizing tokens they had encountered a few times during training. This generalization was less effective in people with DD. Both groups were less accurate in deciphering the trained tokens when these were presented by a new speaker, but on average the typical readers performed better on this test. Thus, there were limits on generalization not only in the DD group but also in the TD group.

One should note that in many laboratory paradigms that address skill acquisition, such as category learning [66] and specifically artificial grammar learning [67], participants are examined on novel (i.e., generalization conditions) items with structural elements similar to those governing the trained set of items or random structures. A similar approach is assumed in some sequence learning paradigms, specifically the serial reaction time task (e.g. the SRT task, [8]) wherein the major condition for learning is the practice-dependent difference that evolves between performance of the repeated sequence versus performance of a random or novel sequence [68]. These test conditions can be viewed as tests of the ability to generalize to a new (yet nevertheless specific) condition, rather than of skill acquisition per se (proficiency, how to knowledge). Thus, some of the inconsistencies in results concerning skill acquisition in DD may relate to the ambiguity of whether the condition used to assess ability and skill acquisition is a transfer test condition or whether it directly reflects intrinsic gains in the performance of the trained condition (for example, performance improvements relative to the initial, pre-training, level). This distinction is quite standard in studies of perceptual learning [59, 61].

The current results suggest that after practice individuals with DD 1) improve no less and perhaps even more than their peers in the acquisition of skills related to the trained (specific, repeated) items; 2) may have equal difficulty (and thus do not differ from typical readers) in generalizing to some new conditions (deciphering trained items presented in a new speaker’s voice); but 3) have specific difficulties in different generalization conditions (deciphering new tokens uttered by the voice heard in training). Thus, the gains achieved by TD and DD groups under identical training conditions may be of similar magnitude relative to the specific training condition and items, yet the two groups may differ in terms of their respective ability to transfer these gains to (some) untrained conditions and items.

Using the logic underlying the analyses of transfer limitations in non-language-related perceptual learning paradigms (e.g, [7, 69]) the differential difficulty in deciphering novel speech items uttered by the trained voice versus the generalizing of performance gains to a new speaker—seen even in typical readers—can be considered as reflecting the processes and perhaps the level of stimulus representation affected by the training experience. One issue is the involvement of declarative memory processes in learning to decipher time-compressed speech. Participants may have formed memory representations for specific items (although the number of token repetitions was quite small, some target sentences were repeated up to four times in the training and pre-test list) that supported their recognition under adverse listening conditions [70]. In the new token condition, however, such memory processes are unlikely to explain the resulting deciphering skill as participants encountered the sentences for the first time (as in the pretest phase). Nevertheless, post-training performance on these novel sentences was superior to naïve performance with time-compressed speech. On the other hand, both groups—TD and DD—had relative difficulty in deciphering the trained tokens when presented by a new speaker’s voice after the training session. This difficulty suggests a practice-dependent reliance on a representation wherein the fundamental frequency of the speaker’s voice is differentially represented. The feature dependency of skills (i.e., specificity of the acquired skill for physical attributes of the trained stimuli) is well recognized in perceptual learning and has been explored in multiple domains [7, 59, 71].

Experiments with speaker variability, speech rate and perceptual learning provide strong evidence for implicit memory for very fine perceptual details of speech (e.g., [71]). Listeners apparently encode specific attributes of the speaker’s voice and speaking rate into long-term memory [72]. Pisoni (67) has suggested that information that is not typically considered to be stored as part of the phonetic or lexical representation of words is nevertheless retained in long-term memory (for example, information about the speech rate or the speaker’s dialect). By this account, specific “episodes” (i.e., instances in which the stimulus was encountered in the input) as well as the operations used for perceptual analysis are encoded and form the foundation for feature-specific (e.g., speaker- or voice-specific) perceptual operations that are part of the procedural memory emerging from the episode (e.g., [73]). Thus, in line with Pisoni’s suggestion (67), the procedures or perceptual operations used to recognize specific, and importantly repeated, speech may generate procedural knowledge, i.e., a processing routine set and honed to accommodate novel and more demanding listening conditions (that have been repeatedly encountered), so that the perceptual analysis for novel words produced by familiar speakers can be carried out efficiently, without the repeated need for detailed analysis of the speaker’s voice [1]; For a similar notion in the visual and motor domain, see [74, 75]. Hence, repeated perceptual episodes (exemplars) with a given speaker’s voice tend to be stored in memory in a feature-specific manner rather than as a general routine [73, 76–78].

The reduced ability of people with DD to transfer their robust practice-related gains when new tokens were presented in the trained voice can indicate that the setting of a processing routine is more heavily weighted for specific items repeatedly encountered in training [78, 79]. The data are also compatible with the notion that people with DD establish processing routines that are more heavily weighted for low-level (feature-specific) representations in deciphering time-compressed speech (as suggested by [71]) This notion is in line with the finding that those in the DD group had more difficulty than their typical reading peers in deciphering familiar tokens presented in the voice of a new (unfamiliar) speaker. Thus, a parsimonious conjecture would be that dyslexics may tend to over-rely on lower-level representations of distorted, unfamiliar speech input. That is, dyslexic readers may be more prone than their normal reading peers to generate feature-specific (and item-specific) routines when provided with repeated experience with challenging input or perhaps they rely more heavily on lower-level skills when faced with perceptually taxing conditions [80]. Thus, the current findings extend previous findings and underscore the notion that generalization problems can affect the ability of people with DD to acquire abstract knowledge from limited experience [29, 81]. Given this notion, perhaps different or modified training conditions are needed when designing learning opportunities for atypical populations to accommodate the different learning strategies of impaired readers. A similar notion has been suggested recently for other special populations as well [82, 83].

Speech perception problems have been documented in people with DD (Rosen, 2003). Most studies, however, assessed the end product of learning (discrimination between phonological categories) [43, 84] rather than the learning process itself [21]. The current results indicate that adaptive training may resolve some of the initial differences observed between TD and DD listeners when deciphering time-compressed speech. This suggests that DD individuals are capable of benefiting from repeated exposure. Yet even after the brief training experience, the ability to generalize what has been learned to new items was more difficult for those with DD. Impaired generalization may have consequences for the ability of those with DD to form "abstract" knowledge. This could explain the difficulties people with DD may have in adjusting to and generalizing some of the variability that characterizes phonological categories.

## Supporting information

S1 File Sample of sentences used in the study. (RAR)
